# A PCR-lateral flow immunochromatographic assay (PCR-LFA) for detecting *Aristolochia* species, the plants responsible for aristolochic acid nephropathy

**DOI:** 10.1038/s41598-022-16528-1

**Published:** 2022-07-16

**Authors:** Kannika Thongkhao, Chayapol Tungphatthong, Suchada Sukrong

**Affiliations:** 1 Center of Excellence in DNA Barcoding of Thai Medicinal Plants, Chulalongkorn University, Bangkok, 10330 Thailand; 2 Department of Pharmacognosy and Pharmaceutical Botany, Faculty of Pharmaceutical Sciences, Chulalongkorn University, Bangkok, 10330 Thailand

**Keywords:** DNA, PCR-based techniques

## Abstract

Aristolochic acids (AAs), which are strong carcinogens, have caused dietary supplements with *Aristolochia* plants to be discontinued worldwide. Therefore, the development of a method to identify these herbs is critical for customer safety. To support the regulation of *Aristolochia*-free products, a PCR coupled with lateral flow immunochromatographic assay (PCR-LFA) that is specific to the nucleotide signature in plastid *rbc*L gene region of *Aristolochia* species was developed to detect *Aristolochia* plants and related herbal products. Triplex primers (A397F, C357F and R502) were designed based on specific nucleotides observed exclusively in the *rbc*L sequences of *Aristolochia*. Positive results for *Aristolochia* occur when the three pink lines are clearly developed on the developed lateral flow strip and can be seen by the naked eye. In this study, the lateral flow strip has sensitivity for detecting amplicons amplified from genomic DNA at the concentrations as low as 0.01 ng. Various kinds of samples, including purchased crude drugs and polyherbal samples, have been investigated, and the results showed that *Aristolochia* crude drugs and *Aristolochia*-containing products are still present in dispensaries. In conclusion, with the goal of protecting consumers from the health risks associated with *Aristolochia* contamination, PCR-LFA was developed and demonstrated to be efficient for detecting plants belonging to *Aristolochia* in various kinds of samples.

## Introduction

**Figure 1:**
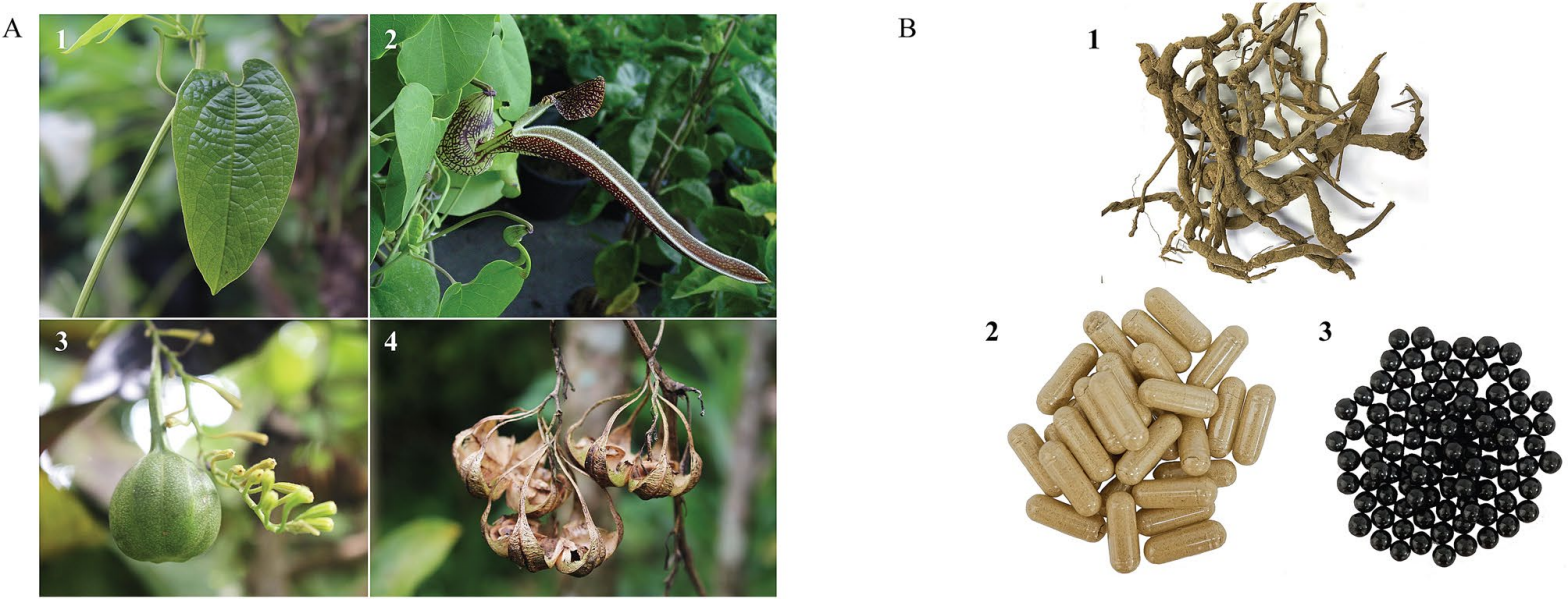
*Aristolochia* plant and herbal products. (**A**) *Aristolochia* plant; **1**: heart-shaped leaf, **2**: flower, **3**: infructescence, **4**: dry pods, (**B**) traditional herbal products; **1**: Krai-Krue crude drug, **2**: capsules of polyherbal formula, **3**: herbal pills. Figure 1A courtesy of Assist. Prof. Aekkhaluck Intharuksa.

Plants belonging to the genus *Aristolochia* (Aristolochiaceae) are distributed in high temperature and tropical climates^1^ (Fig. 1). There are approximately 500 *Aristolochia* species worldwide^2^. *Aristolochia* plants have stems that are erect or twining^3^. Leaves are alternate and cordate with unique pipe-shaped inflorescences and fruits are dehiscent capsules (Fig. 1A). Several *Aristolochia* species have been medically used for the treatment of diseases and illness. For example, *Aristolochia fangchi* Y.C. Wu ex L.D. Chow & S.M. Hwang has been applied for antirheumatic and diuretic care in China. Traditional healing practitioners in India use *A. indica* L. as antivenom and skin diseases. Based on Sudan’s folk medicine, *A. bracteolata* Lam. is applied for infectious diseases treatment^4^. In Thailand, *A. acuminata* Lam. (also known as *A. tagala* Cham.) is reported to treat viral and helminth worm infections in northern and western regions^5^. Thai traditional medicine (TTM) utilizes *A. acuminata* as an antipyretic, anti-inflammatory agent, muscle relaxant, appetite-enhancing agent, and analeptic^6^. *A. pierrei* Lecomte, *A. pothieri* Pierre ex Lecomte and *A. acuminata*, named “Krai-Krue” in Thai, are used as ingredients in TTM formulae (Fig. 1B) to treat dizziness, nausea, vomiting, flatulence, and cough relief^7^. Besides the use of *A. pierrei*, *A. pothieri* and *A. acuminata* as “Krai-Krue” crude drug, their substitute species; *Cynanchum pulchellum* (Wall.) Liede & Khanum, *Trichosanthes scabra* Lour., *Jasminum sambac* (L.) Aiton, *J. adenophyllum* Wall. ex C.B. Clarke. have been reported^8^.

In addition to the healing properties of *Aristolochia* herbs as mentioned, plants in the Aristolochiaceae family including *Aristolochia, Asarum, Saruma,* and *Thottea* genera contain aristolochic acids (AAs), which cause aristolochic acid nephropathy (AAN) and end-stage renal failure associated with urothelial carcinoma^9–11^. After the carcinogenic and mutagenic properties of AAs were reported, several countries and regions have taken different regulations and measures to address the issue. In 1994, the French Ministry of Health banned the sale of drugs containing AA and the British Committee on Safety of Medicines (CSM) banned the use of botanical drugs containing AA in 1999^12^. In 2001, all manufacturers in the USA were urged by the United States Food and Drug Administration (USFDA) to ensure AA-free herbal products; moreover, the use of AA-containing products was stopped^13^. The use of *Aristolochia* plants as traditional medicine materials was discontinued in Europe, the USA, Australia, and Asia^11, 14^. In 2002, the World Health Organization International Agency for Research on Cancer (WHO IARC) classifies AAs as carcinogen^15^. Since 2011, the National Drug Committee of Thailand legally announced the withdrawal of *Aristolochia* herbs from the recipes of TTM formulae, including “Ya Tad Ban Job (YTBJ)”, “Ya Um Ma Rue Ka Va Tee (YMVT)” and “Ya Pra Sa Jet Ta Pung Ki (YPSJ)”^7^. Although *Aristolochia* has been banned and regulated, AA-containing products are still sold in markets ^16^. This is due to the belief on the inexplicable TTM that a very small dose of toxic substances can be neutralized by the other compounds in the herbal formula^17^. To date, there is an increasing number of AAN patients who have consumed botanical products ^11^.

To control quality of herbal drugs, the Thai Herbal Pharmacopoeia (THP) 2021 established monographs of *Aristolochia* crud drugs to provide quality control standard guideline for manufacturers to ensure that their products are free of AAs^18^. *Aristolochia* detection methods are mainly focused on AAs and their derivatives using a variety of techniques, such as high-performance liquid chromatography (HPLC), mass spectrometry (MS), liquid chromatography mass spectrometry (LC–MS), nuclear magnetic resonance (NMR) and high-performance thin-layer chromatography (HPTLC)^19–21^. Among the previous mentioned analytical methods, the HPLC and thin-layer-chromatography were issued in the American Herbal Pharmacopoeia (AHP)^22^ and THP 2021, respectively. Although the previously mentioned analytical methods are robust, efficient, and sensitive, they are time-consuming, laborious, and complicate^23^. Furthermore, the phytochemical compounds of herbs may vary with age, harvest time and geographical differences^24^. Thus, employing only chemicals for plant authentication is difficult. Alternatively, DNA information does not vary by environmental factors. Using nucleotide differences among species is an easy and reliable technique to differentiate targeted species from others^25^. The nucleotide signature at the *rbc*L DNA region, one of the core DNA barcodes including internal transcribed spacer (ITS), maturase K (*mat*K) and *psb*A-*trn*H intergenic spacer regions that is unique to a specific species or genus, is an effective molecular tool for assisting species identification^8, 26^. Quantitative real-time PCR (qPCR), one of the sensitive DNA-based technique has been used for quality control testing in herbal industry^27^. It is simple, fast and sensitive test; however, it requires standard curve from known quantities of the target. In 2018, Sgamma et al., developed a high sensitivity method in detection of *Aristolochia* contamination in medical plants materials using quantitative real-time PCR (qPCR) coupled with specific primers^28^.

Currently, polymerase chain reaction coupled with a lateral flow immunochromatographic assay (PCR-LFA) enables targeted DNA to be sensitively detected without using agarose gel electrophoresis, therefore the species analysis procedure becomes more rapid and easier^29^. Rapid authentication of species by species-specific sequence coupled with LFA has been developed for the identification of many living species, including *Ophiocordyceps sinensis*^24^, *Cannabis* spp.^30^ and *Salmonella* spp.^31^ Expert personnel or expensive equipment are no longer required; moreover, the results can be simply inspected by the naked eye. Thus, we aimed to develop a PCR-LFA based on the nucleotide signature of the *rbc*L region to detect *Aristolochia,* the plants responsible for aristolochic acid nephropathy. PCR-LFA was used to investigate the suspected *Aristolochia*-containing herbal formulae and crude drug samples from various local dispensaries in Thailand.

## Result

### *Aristolochia*’s nucleotide signature on the *rbc*L gene and the development of PCR-LFA

**Figure 2:**
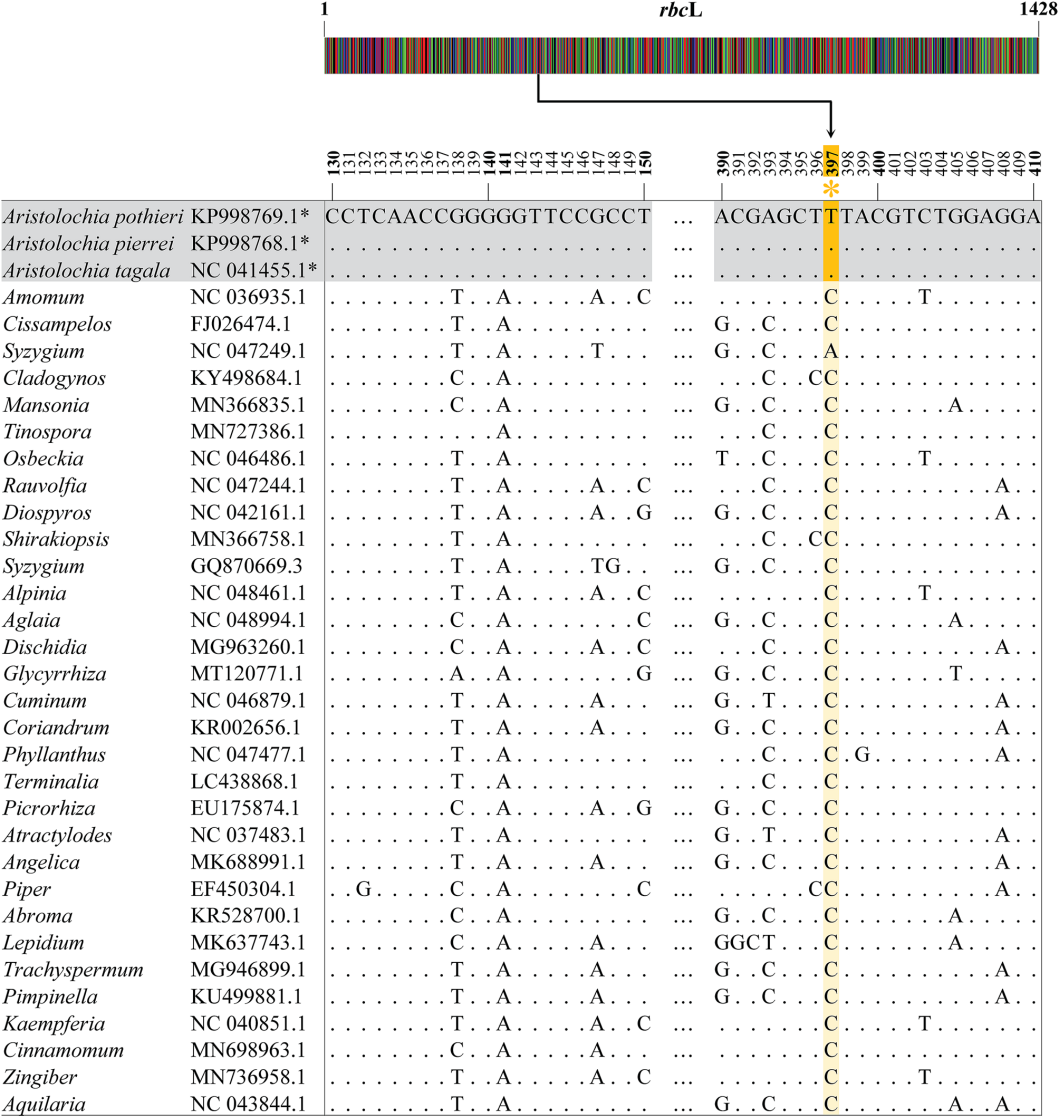
Alignment of the partial *rbc*L sequences of the selected *Aristolochia* spp. and non-*Aristolochia* plants. The nucleotide signature of *Aristolochia* plants is colored yellow. Asterisks indicate nucleotide signatures that target the specific primer. Consensus sequences are indicated with dots. The altered bases indicate the sequence differences.

**Figure 3:**
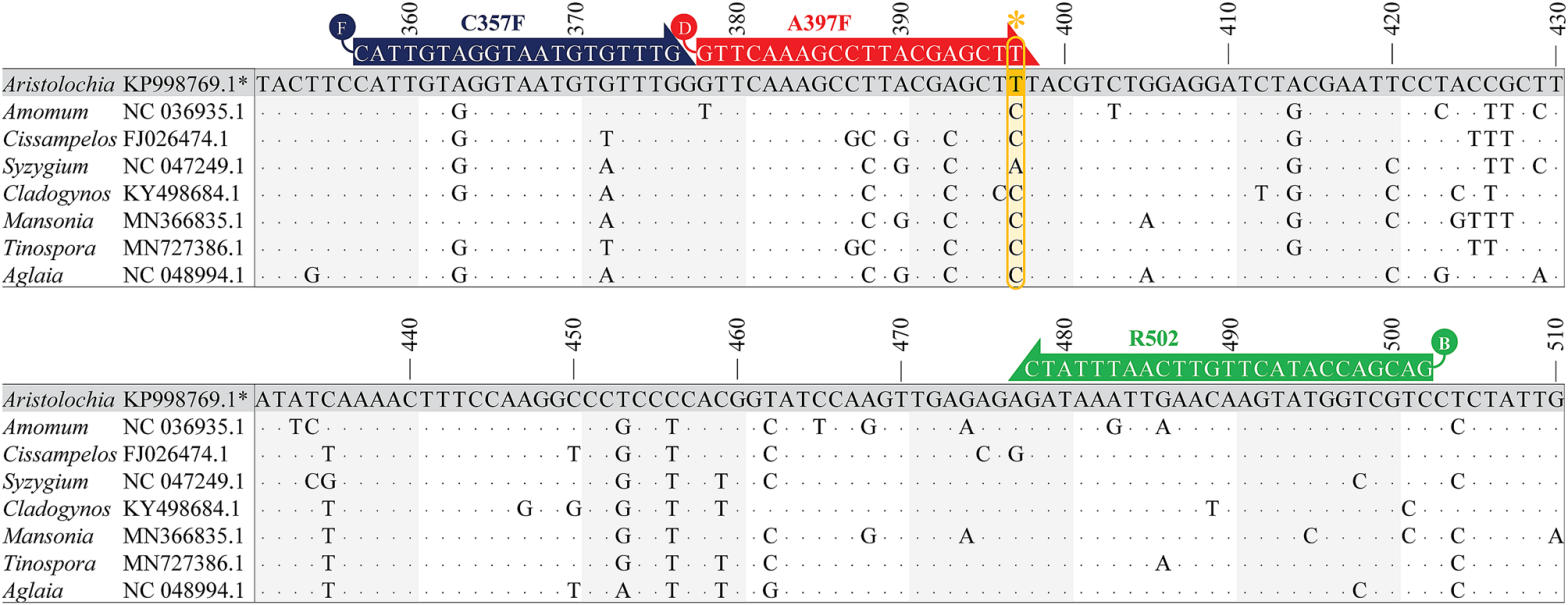
The primer design for the detection of *Aristolochia* spp. by PCR*-*LFA on a partial *rbc*L gene alignment. Selected plant species are shown in the alignment. Primers A397F (red) and R502 (green) were used for amplification of the internal control amplicon. Primer C357F (blue and R502 (green) was used for amplification of the *Aristolochia*-specific PCR amplicon. At the 5'-end of C357F, R502 and A397F primers were labeled with FAM (F), biotin (B) and DIG (D), respectively. The yellow box and asterisk present the nucleotide signature of the *Aristolochia* plant. Consensus sequences are indicated with dots. The altered bases indicate the sequence differences.

*Rbc*L gene sequences of 111 selected accession numbers belonging to 30 plant genera that are often used as herbal ingredients along with *Aristolochia* plants used in TTM formulae were retrieved from GenBank (Supplementary Table S1). The sequences differed in length, ranging from 300 to 1464 bp. From the DNA alignment (Supplementary Table S2), nucleotide “T” at position 397 was unique to the *Aristolochia* genus, while other non-*Aristolochia* plants used exhibited C or A (Fig. 2). Based on the unique nucleotide 397 T of the *rbc*L gene of *Aristolochia* spp., a 20 bp *Aristolochia*-specific primer (A397F) was designed for the amplification of genomic DNA from *Aristolochia* plants. Blast results of our designed primer A397F (GTTCAAAGCCTTACGAGCTT) against the NCBI database showed 100% identity to plant species, *Aristolochia.* (Supplementary Fig. S1). Internal control primers, C357F and R502 primers at positions 357–376 and 478–502 of the *rbc*L gene, respectively, were designed based on conserved sequences of 177 accession numbers. To detect the amplicons by the PCR-LFA system, all primers were tagged on the 5′ ends by different antigens. In this study, C357F, R502 and A397F primers were labeled with fluorescein (FAM), biotin, and digoxigenin (DIG) (Fig. 3). Duplex PCR amplification using A397F, C357F and R502 primers generated two slightly different amplicons, including *Aristolochia*-specific amplicons (amplified by A397F and R502 primers) and internal control amplicons (amplified by C357F and R502 primers), at 124 bp and 145 bp, respectively (Fig. 3).

**Figure 4:**
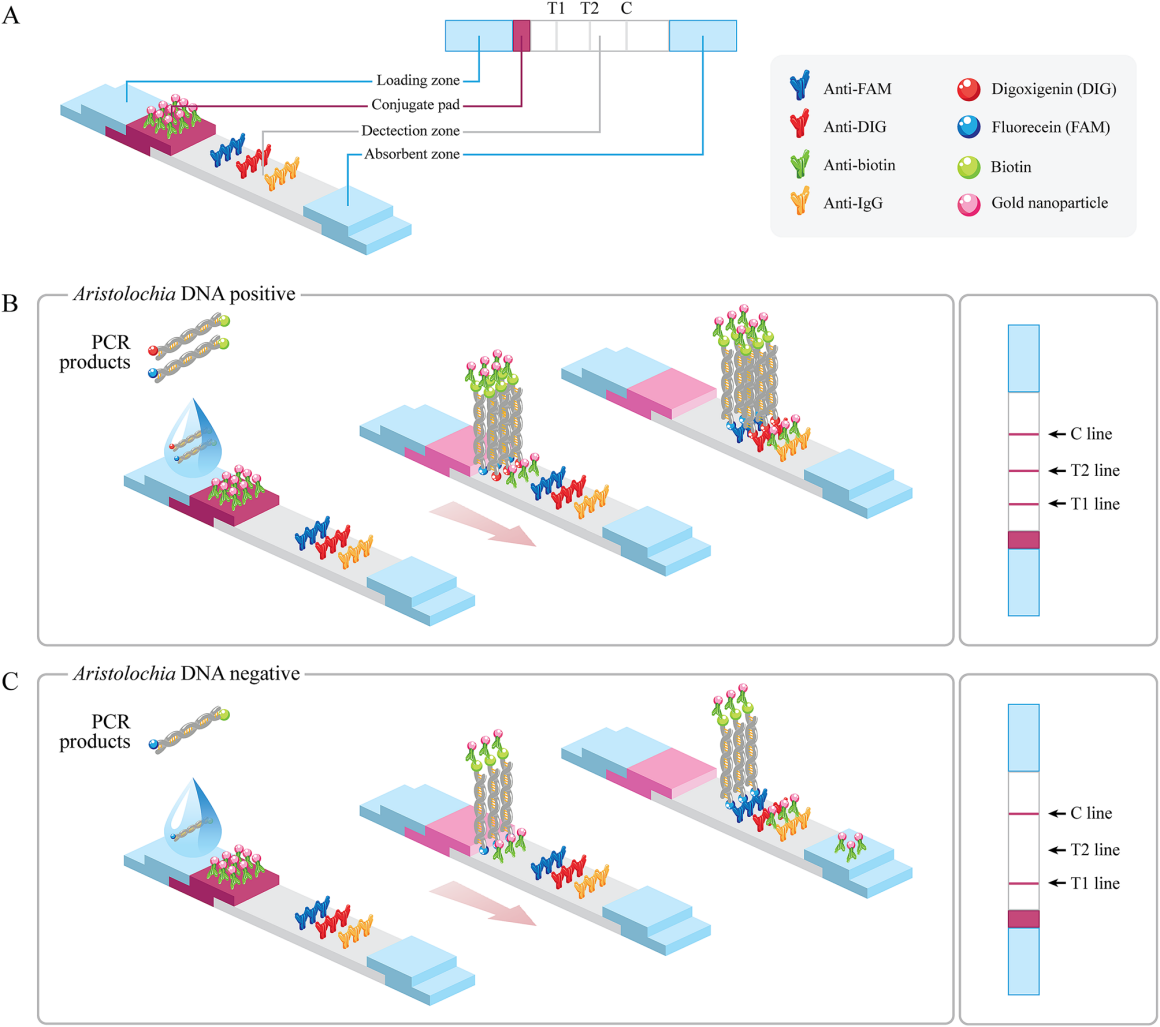
Principle of PCR-LFA to detect *Aristolochia* spp. (**A**) The following four elements of the LFA strip were used: loading zone, conjugate pad, detection zone and adsorbent pad with immobilized antibodies (anti-FAM, anti-DIG and anti-IgG) and adsorbent pad. (**B**) *Aristolochia-*LFA positive result, dual labeling of the internal control amplicon and the *Aristolochia*-specific amplicons bound to the antibiotin-gold nanoparticle conjugates at the conjugate pad and migrated through the detection zone. The complexes containing FAM and DIG were trapped by their specific antibodies at the detection zone, creating coloration at both the T1 and T2 lines. Unbound was captured by anti-IgG at the C line, (**C**) *Aristolochia-*LFA negative result, the dual labeling of internal control amplicon was migrated along the detection zone and pink color were observed at T1 and C lines but not T2 line.

The LFA strip was composed of a nitrocellulose membrane that was 3 mm wide and 8 cm high. The strip included a loading zone, conjugate pad, detection zone and absorbent pad. Gold nanoparticle (GNP)-conjugated antibiotin was coated on a conjugate pad (Fig. 4). In the detection zone, an internal control line (T1 line), *Aristolochia* line (T2 line), and strip control line (C line) were created by immobilizing an anti-FAM capture antibody, anti-DIG capture antibody and anti-mouse IgG antibody, respectively (Fig. 4A). In the LFA system, a PCR control line (T1 line) with pink color should be clearly observed in all tested samples (Fig. 4B and C) except for the reaction without the DNA template. Pink was also present on the *Aristolochia* line (T2 line) in all *Aristolochia* plants (Fig. 4B) but absent in non-*Aristolochia* plants used (Fig. 4C). A C line is observed in all tests.

### Specificity and sensitivity of the PCR-LFA

**Figure 5:**
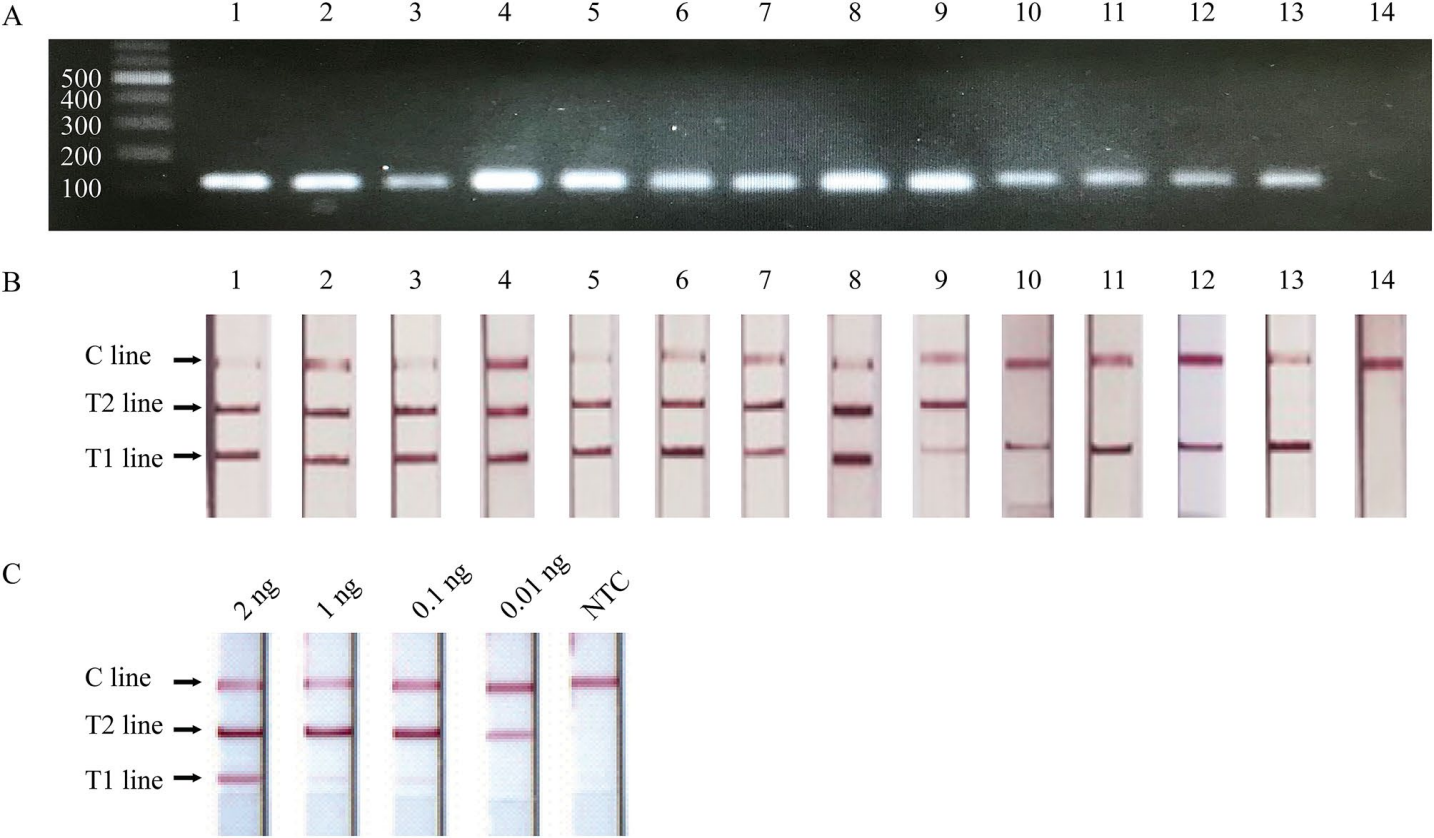
Specificity and sensitivity of the PCR*-*LFA for the detection of PCR amplicons generated with a set of primers, A397F, C357F and R502. (**A**) Image of PCR amplicons as detected by 1.7% agarose gel electrophoresis. **1**: *A. pierrei*, **2**: *A. pothieri*, **3**: *A. acuminata*, **4**: *A. gigantea.* **5**: *A. grandiflora*, **6**: *A. cambodiana*, **7**: *A. littoralis,* **8**: *A. ringens*, **9**: *A. tentaculata*, **10**: *C. pulchellum*, **11**: *T. scabra*, **12**: *J. sambac*, **13**: *J. adenophyllum*, **14**: no-DNA template as a control reaction. (**B**) T2 line was detected from *Aristolochia* spp. samples (1–9) but not detected from non-*Aristolochia* samples (10–14). (**C**) Sensitivity of the *Aristolochia* PCR*-*LFA. Template DNA: 2, 1, 0.1 and 0.01 ng. **NTC**: no-DNA template.

To evaluate the specificity of this lateral flow system for *Aristolochia* detection, plant samples, including *Aristolochia* and non-*Aristolochia* species (Table 1), were challenged and tested (Fig. 5, Supplementary Fig. S2). The PCR amplicons from all tested samples subjected to agarose gel electrophoresis revealed DNA bands from either *Aristolochia*-specific amplicons or internal control amplicons at 124 bp and 145 bp, respectively (Fig. 5A). The two PCR amplicons with similar sizes, 124 bp and 145 bp in length, were present as one band. By PCR-LFA testing, three lines of T1, T2 and C lines were simultaneously detected in all *Aristolochia* plants (lanes 1–9), including *A. pierrei*, *A. pothieri*, *A.* *acuminata*, *A. gigantea*, *A. grandiflora*, *A. cambodiana*, *A. littoralis*, *A. ringens* and *A. tentaculata,* while T1 and C lines were observed in non-*Aristolochia* plants (lanes 10–13), including *Cynanchum pulchellum* (Wall.) Liede & Khanum, *Trichosanthes scabra* Lour.*, Jasminum sambac* (L.) Aiton*,* and *Jasminum adenophyllum* Wall. ex C.B. Clarke (Fig. 5B). To confirm the absence of false positive results from primer-dimer hybridization, the lateral flow strip was tested with duplex PCR without genomic DNA added (lane 14). The results showed only a single line on line C (Fig. 5A and B). To confirm the positive result from PCR-LFA for the presence of *Aristolochia*, *Aristolochia*-specific amplicons from *A. pothieri* were sequenced, and the result showed the nucleotide identity to the *rbc*L sequence of *Aristolochia* spp. (Supplementary Fig. S3).

**Table 1:** Plant materials used in this study.

Samples	Specimens no	Voucher no	Scientific name	Place of collection (Province in Thailand)
**Plant samples**				
*Aristolochia* spp.	SS-A0715	MUS-5400	*Aristolochia acuminata* Lam	Chiang Mai
	SS-A0716	MUS-5407	*A. pierrei* Lecomte	Sakon Nakhon
	SS-A0717	MUS-5374	*A. pothieri* Pierre ex Lecomte	Bangkok
	SS-A0718	MUS-5393	*A. gigantea* Mart. Et Zucc	Bangkok
	SS-A0719	MUS-5391	*A. grandiflora* Sw	Lampang
	SS-A0720	MUS-5413	*A. cambodiana* Pierre ex	Chiang Mai
	SS-A0721	MUS-5404	*A. littoralis* Parodi	Bangkok
	SS-A0722	MUS-5375	*A. ringens* Vahl	Bangkok
	SS-A0723	MUS-5398	*A. tentaculata* O.C.Schmidt	Bangkok
Non-*Aristolochia* spp.	SS-A0724	MUS-5414	*Cynanchum pulchellum* (Wall.) Liede & Khanum	Bangkok
	SS-A0725	MUS-5415	*Trichosanthes scabra* Lour	Sakaeo
	SS-A0726	MUS-5417	*Jasminum sambac* (L.) Aiton	Bangkok
	SS-A0727	MUS-5418	*Jasminum adenophyllum* Wall. ex C.B.Clarke	Bangkok
	SS-A0728	MUS-6001	*Terminalia chebula* Retz	Bangkok
	SS-A0729	MUS-6002	*Glycyrrhiza glabra* L	Bangkok
	SS-A0730	MUS-6003	*Cuminum cyminum* L	Bangkok
	SS-A0731	MUS-6004	*Coriandrum sativum* L	Bangkok
	SS-A0732	MUS-6005	*Phyllanthus emblica* L	Bangkok
	SS-A0733	MUS-6006	*Terminalia bellirica* (Gaertn.) Roxb	Bangkok
**Crude drugs claimed to be Aristolochia?**				
Crude drug 1	C1	CMUS-2022	**–**	Bangkok
Crude drug 2	C2	CMUS-2023	**–**	Bangkok
Crude drug 3	C3	CMUS-2024	**–**	Nakhon Si Thammarat
Crude drug 4	C4	CMUS-2025	**–**	Phetchaburi
Crude drug 5	C5	CMUS-2026	**–**	Ayutthaya
Crude drug 6	C6	CMUS-2027	**–**	Bangkok
Crude drug 7	C7	CMUS-2028	**–**	Bangkok
Crude drug 8	C8	CMUS-2029	**–**	Bangkok
**Purchase polyherbal samples**				
Laboratory-made sample (YMVT)	F0	FMUS-0127	**–**	Bangkok
Herbal formula 1	F1	FMUS-0123	**–**	Bangkok
Herbal formula 2	F2	FMUS-0124	**–**	Bangkok
Herbal formula 3	F3	FMUS-0125	**–**	Bangkok
Herbal formula 4	F4	FMUS-0126	**–**	Bangkok

Different amounts of total genomic DNA (2, 1, 0.1 and 0.01 ng) from *Aristolochia* plants were examined to assess the sensitivity of our developed PCR*-*LFA (Fig. 5C). The T1 line of the internal control amplicon was observed with a slight gradient of pink color from dark to faint at all DNA concentrations from 2 to 0.01 ng. The lateral flow strip has good sensitivity for the detection of *Aristolochia*-specific amplicons from DNA at a concentration as low as 0.01 ng, as the T2 line was very clearly observed. The C line was detected at all DNA concentrations, including the no-DNA template control reaction (NTC).

### Utilization of the PCR-LFA for examining herbal samples

**Figure 6:**
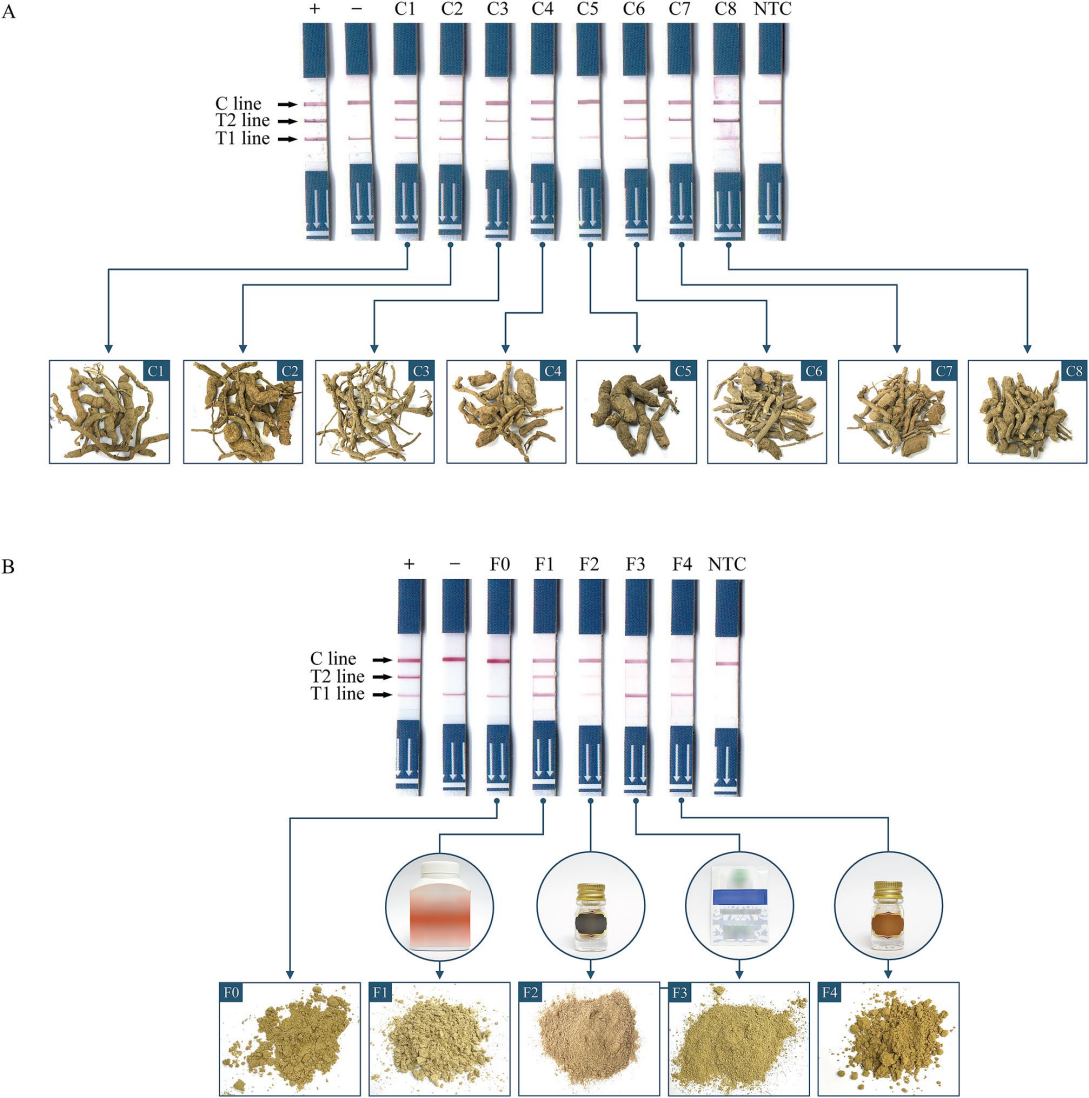
Testing the existence of *Aristolochia* spp*.* in herbal products with the PCR- LFA method. (**A**) crude drugs and (**B**) herbal formulae. + : positive control, − : negative control, C1-C8: crude drug no. 1–8, F0: laboratory-made formulae (YMVT), F1-4: herbal formula no. 1–4, NTC: no-DNA template as a control reaction.

The PCR*-*LFA method was used to test the botanical origins of commercial crude drugs and herbal formulae purchased from Thai local shops (Table 2). The genomic DNA from crude drugs was extracted, amplified, and subjected to PCR-LFA, and the results showed the presence of the T1 line in all samples (Fig. 6, Supplementary Fig. S4). The T2 line was observed in seven (C1, C2, C3, C4, C6, C7 and C8) out of eight crude drug samples (C1-C8), while it was absent in C5 (Fig. 6A). In gel electrophoresis, the PCR amplicon obtained from the duplex PCR using A397F and R502 primers revealed no *Aristolochia*-specific amplicon from the C5 samples (the data are not shown). C7 and C8 samples were selected as two representatives that were further confirmed by duplex PCR amplification to obtain specific amplicons. The amplicons were 124 bp in length, and the subsequent sequencing results showed that the nucleotide at position 397 was “T” (Supplementary Fig. S5).

**Table 2:** Detection results of the tested samples by the PCR-LFA.

Samples	Scientific Name/Sample Name	T1 line	T2 line	C line	Detected species
*Aristolochia* species	*A. pierrei* Lecomte	++	++	++	*Aristolochia* sp.
	*A. pothieri* Pierre ex Lecomte	++	++	++	Aristolochia sp.
	*A. acuminata* Lam	++	++	++	*Aristolochia* sp.
	*A. gigantea* Mart. Et Zucc	++	++	++	*Aristolochia* sp.
	*A. grandiflora* Sw	++	++	++	*Aristolochia* sp.
	*A. cambodiana* Pierre ex	++	++	++	*Aristolochia* sp.
	*A. littoralis* Parodi	++	++	++	*Aristolochia* sp.
	*A. ringens* Vahl	++	++	++	*Aristolochia* sp.
	*A. tentaculata* O.C.Schmidt	++	++	++	*Aristolochia* sp.
Non-*Aristolochia* species	*Cynanchum pulchellum* (Wall.) Liede & Khanum	++	–	++	Non-*Aristolochia* sp.
	*Trichosanthes scabra* Lour	++	–	++	Non-*Aristolochia* sp.
	*Jasminum sambac* (L.) Aiton	++	–	++	Non-*Aristolochia* sp.
	*Jasminum adenophyllum* Wall. ex C.B.Clarke	++	–	++	Non-*Aristolochia* sp.
Crude drugs	C1	+	++	++	*Aristolochia* sp.
	C2	+	++	++	*Aristolochia* sp.
	C3	+	++	++	*Aristolochia* sp.
	C4	+	++	++	*Aristolochia* sp.
	C5	+	–	++	Non-*Aristolochia* sp.
	C6	+	++	++	*Aristolochia* sp.
	C7	+	++	++	*Aristolochia* sp.
	C8	+	++	++	*Aristolochia* sp.
Herbal formulae	F0 (YMVT)	++	–	++	Non-*Aristolochia* sp.
	F1	++	++	++	*Aristolochia* sp.
	F2	+	+	++	*Aristolochia* sp.
	F3	++	–	++	Non-*Aristolochia* sp.
	F4	++	+	++	*Aristolochia* sp.

Authentic *Aristolochia* spp. and selected plants were challenged with the PCR-LFA method.

++: clearly pink color.

+ : faint pink color.

–: coloration was not observed.

For the applicability of the PCR*-*LFA in herbal-mixed formulae, the selected laboratory-made “YMVT” and commercial polyherbal samples were challenged (Table 2 and Fig. 6B). For YMVT (F0; free of *Aristolochia*), the results showed that T1 and C lines were present but not the T2 line. For the commercial polyherbal samples that were tested, the T1 and C lines appeared in all samples. Three lines, the T1, T2 and C lines, were observed in F1, F2 and F4. The C and T1 lines were observed from commercial polyherbal F3. PCR amplicons from selected polyherbal F1 sample were chosen for sequencing, and the results revealed *Aristolochia*-specific nucleotides (397 T) and sequences matched to *Aristolochia* plants in the GenBank database (Supplementary Fig. S5).

## Discussion

Although the use of *Aristolochia* as herbal medicine is banned in many countries due to the risks of kidney injuries, cancer, and deaths, case reports with *Aristolochia*-containing products (herbs and dietary supplements) are still being documented. *Aristolochia* plants can be unintentionally substituted in herbal products, which can be caused by misidentification, inappropriate nomenclature, and imprecise labeling^32^. To assist in the identification of *Aristolochia* materials, a number of publications, including species-specific multiplex primers coupled with HPTLC^8^, real-time PCR combined with ultrahigh-performance liquid chromatography–mass spectrometry (UHPLC-HR-MS)^9^, and quantitative real-time PCR^28^, have been reported. Although standard methods for herbal species identification, such as organoleptic, micro- and macroscopic methods and TLC, are recommended in the Pharmacopoeia of many countries, including the Thai Herbal Pharmacopoeia (THP)^33^, utilizing genetic information is still a reliable technique to differentiate targeted species from others. The nucleotide signature, the term that refers to one or more nucleotides unique to one taxon from a specific DNA barcode region, is found to be a very efficient tool for the identification of herbal species and products^34^.

Herbs containing secondary compounds such as polysaccharides, pigments, tannins and resins may affect PCR amplification^35^. In this study, the herbal medicine samples were washed in wash buffer multiple times to remove sticky residues, but some residues may have remained. These residues could interfere with the DNA extraction step and cause the quantity of DNA template to be insufficient for PCR amplification^36^. Moreover, the existing secondary metabolites could also interfere with the PCR amplification process^37^ and subsequently result in a faint pink band on the test lines in PCR-LFA. This situation indicates that PCR-LFA is sensitive enough even in challenging samples. This finding agrees with the fact that LFA is a highly sensitive, simple, fast, cost-effective, and user-friendly detection method that does not require experts^38^.

In our study, an exclusive nucleotide signature “T” at position 397 of the *rbc*L gene of plants in the genus *Aristolochia* was identified. Nucleotides at 397(T) were unique to the *Aristolochia* genus, while other non-*Aristolochia* plants exhibited C or A. Other genera of family Aristolochiaceae i.e. *Asarum*, *Saruma* and *Thottea*, which have been shown to contain AAs^10, 39^, the position 397 of *rbc*L gene exhibited nucleotide C, C and T, respectively (Supplementary Data S1). Our finding agreed with a previous report that *rbc*L has a rare rate of recombination with less intraspecific variation than interspecific variation, which is the key criterion for the use of any DNA sequence as a nucleotide signature^40^. The *rbc*L region has some limitations regarding its ability for species resolution in plants. For example, the gene lacks sufficient sequence variation to distinguish closely related species^41^ and it is also the mononucleotide repeats region^42^, which can generate short sequence during barcoding process. However, it has higher interspecific distance compared to other core DNA barcode regions (*mat*K, ITS and *psb*A-*trn*H), therefore it is beneficial to our study as a candidate region to identify plant materials at the generic level and above. Furthermore, the *rbc*L gene is easy to amplify and provides sequences of good quality compared to other regions^43^.

In this study, sequence-specific PCR primers (primer A397F) were employed with a T at its 3′ end at position 397 of the *rbc*L gene, which amplified only the *Aristolochia*’s DNAs that had a T in this position, while the primer failed to amplify in those of non-*Aristolochia* plants. BLAST result of amplicon generated from A397F and R502 primers showed 100% identity to *rbc*L of plants in *Aristolochia*. This further confirms the specificity of this signature for the genus *Aristolochia*. Among 31 plant genera used in this study, we have not found the T ‘signature’ at position 397 that belong to different families but Aristolochiaceae plants. Interestingly, the alignment of the *rbc*L sequences including plants from the different genera of the Aristolochiaceae family including *Aristolochia*, *Asarum*, *Saruma,* and *Thottea* was performed and found that there was the T ‘signature’ at position 397 in the genus *Thottea*, however, sequence variations near position 397 still were observed among the genus (Supplementary Table S3, Supplementary Data S1). Primer of the A397F was specifically designed to match to *Aristolochia*, therefore, the DNA amplification of *Asarum*, *Saruma* and *Thottea* by using A397F primer would be challenged because of some nucleotide mismatched. The possibility is opened to search for family-specific primers to identify herbal materials from the Aristolochiaceae family containing AA. Based on the nucleotide signature of *Aristolochia*, PCR-LFA was developed and used for the differentiation of *Aristolochia* from other plant species. However, the ability to perform on-site detection with PCR-LFA is limited because a PCR machine is needed. Therefore, a new amplification technique such as recombinase polymerase amplification (RPA) can be applied to support on-site detection, as the method involves isothermal amplification and only a lab heat block is necessary^44^. To further study medicinal ingredients, a promising direction would be to combine the nucleotide signature method with the high-throughput sequencing method.

Our work showed the detection of *Aristolochia* from randomly purchased crude drugs and polyherbal products, which indicated that prohibited plants with the ability of causing nephropathy are available on the market. The presence of *Aristolochia* plants affects consumer safety; therefore, regulatory agencies should be taken into account, and quality control restrictions should be utilized.

## Materials and methods

### Plant materials and a laboratory-made herbal formula

Authentic *Aristolochia* plants; *A. acuminata* Lam., *A. pierrei* Lecomte, *A. pothieri* Pierre ex Lecomte, *A. gigantea* Mart. Et Zucc., *A. grandiflora* Sw., *A. cambodiana* Pierre ex, *A. littoralis* Parodi, *A. ringens* Vahl and *A. tentaculata* O.C. Schmidt were collected from different regions of Thailand. Non-*Aristolochia* herbs that often used as substituted for *Aristolochia* plants under the named “Krai-Krue”; *Cynanchum pulchellum* (Wall.) Liede & Khanum, *Trichosanthes scabra* Lour., *Jasminum sambac* (L.) Aiton, *J. adenophyllum* Wall. ex C.B. Clarke. Non-*Aristolochia* herbs composed of the “YMVT”, including *Terminalia chebula* Retz, *Glycyrrhiza glabra* L., *Cuminum cyminum* L., *Coriandrum sativum* L., *Phyllanthus emblica* L. and *Terminalia bellirica* (Gaertn.) Roxb were obtained from herbal drug stores (Table 1). Those collections are permitted and legal.

Eight crude drugs were purchased from herbal drug stores. Four polyherbal medicine products were bought from online shops and herbal drug stores. The laboratory-made herbal formula “YMVT” was chosen as a representative sample of the mixed formula for this study. It was prepared by mixing *Glycyrrhiza glabra, Terminalia chebula*, *Cuminum cyminum*, *Coriandrum sativum*, and *Phyllanthus emblica* and *Terminalia bellirica* (mixing ratio: 35:7:7:7:7:7) as described in the Thailand National List of Essential Medicines (NLEM).

All species were authenticated by a taxonomist, Associate Professor Thatree Phadungcharoen at the Department of Pharmacognosy and Pharmaceutical Botany, Faculty of Pharmaceutical Sciences, Chulalongkorn University, Thailand. Voucher specimens from all plants used in this study were assigned and kept at the Museum of Natural Medicine, Faculty of Pharmaceutical Sciences, Chulalongkorn University, Thailand. All the experiments were performed in accordance with relevant guidelines and regulations.

### DNA extraction

Fresh leaves of authentic *Aristolochia* plants were ground into fine powder with a mortar and pestle in liquid nitrogen. Genomic DNA was extracted from 50 mg of fine powder using a DNeasy Plant Mini Kit (Qiagen, Germany) following the manufacturer’s instructions. A GENECLEAN Kit (MP Biomedicals, USA) was used to purify the genomic DNA according to the manufacturer’s protocol. The quantity of the extracted DNA was determined spectrophotometrically using a NanoDrop One UV–Vis Spectrophotometer (Thermo Scientific, USA). DNA quality was observed by agarose gel electrophoresis. Genomic DNA was run on 0.8% (w/v) agarose in 1X TAE gel containing 1X RedSafe nucleic acid staining solution (iNtRON Biotechnology, USA) at 100 V for 30 min. The agarose gel was analyzed with a UVP GelSolo (Analytik Jena GmbH, Germany) gel documentation system. Images were taken by onboard VisionWorks software (Analytik Jena GmbH, Germany). Genomic DNA was stored at − 20 °C for further use.

For crude drugs and herbal medicine formulae, the herbal products (30–50 mg) were crushed into fine powder and washed in prewash buffer (100 mM Tris–HCl pH 8.0, 20 mM EDTA, 700 mM NaCl, 4% PVP and 0.4% β-mercaptoethanol) until a clear solution was observed (approximately 5–6 times). The additional wash step was modified from the prewash method published by Xin et al.^45^ Genomic DNA was extracted and purified using the methods described above. Genomic DNA quantification and qualification were conducted as described for the authentic plant samples. Genomic DNA was stored at − 20 °C for further use.

### Sequence analysis of the *rbc*L region

A nucleotide sequence dataset of the *rbc*L region of the 31 plant species listed in the three THM recipes, YTBJ, YPSJ and YMVT, in a total of 111 accession numbers was retrieved from GenBank (Supplementary Table 1). The dataset contained 66 and 111 accession numbers of *Aristolochia* spp. and non-*Aristolochia* plants, respectively (Supplementary Table 2). All sequences were aligned using MEGA X: Molecular Evolutionary Genetics Analysis across computing platforms (MEGA X) version 10.1 with gap open =  − 400; gap extend = 0; clustering method = UPGMA; and Min Diag Length = 24. Nucleotide polymorphisms were determined. Specific nucleotide signatures that differentiated *Aristolochia* plants from other plants were identified.

### Primers for PCR-LFA

Based on the alignment of the *rbc*L gene obtained from *Aristolochia* and non-*Aristolochia* plants, a primer set for PCR-LFA was designed that consisted of an *Aristolochia*-specific primer (A397F at position 378–397) and two internal control primers (C357F and R502 at positions 357–376 and 478–502, respectively) (Fig. 2). An *Aristolochia*-specific primer (A397F) was designed based on specific nucleotides of *Aristolochia* plants in the *rbc*L region. The A397F primer was blasted against the NCBI database to check for robust specificity to *Aristolochia*. The 3ʹ-end of A397F was specifically located on the nucleotide signature of *Aristolochia* plants. The *Aristolochia*-specific amplicon was amplified by using A397F and R502 primers. An internal control amplicon (C357F and R502 primers) was designed based on conserved sequences of 177 accession numbers that were retrieved from GenBank. Each primer was tagged with three different labeling molecules. Digoxigenin (DIG) was attached at the 5ʹ-end of A397F ([DIG]-GTTCAAAGCCTTACGAGCTT). The 5ʹ-end of C357F was labeled with a 5′ 6-fluorescence (FAM) ([FAM]-CATTGTAGGTAATGTATTTG). Biotin was attached at the 5ʹ-end of R502 ([Biotin]-GACGACCATACTTGTTCA ATTTATC) (Fig. 2).

### PCR-LFA

Multiplex PCR mixtures were conducted in a 20 µL total reaction. The mixture contained 10 ng DNA template, 1X Platinum *Taq* PCR buffer, 3 mM MgCl_2_, 200 µM dNTPs, primer set containing 0.25 µM of each primer (A397F, C357F and R502) and 0.25 U of Platinum *Taq* DNA polymerase (Invitrogen, USA). PCR amplification was carried out in a T100 Thermal Cycler (Bio–Rad, USA) using cycling conditions of 94 °C for 4 min, which was followed by 30 cycles of 94 °C for 30 s, 53 °C for 20 s, and 72 °C for 30 s; then, a final extension was performed at 72 °C for 5 min.

To test the present of *Aristolochia* spp. in crude drugs and polyherbal products, dual-labeled amplicons amplified by A397F, C357F and R502 primers were detected by the commercialized PCR-LFA strip (Lot. No. 20F0509, Kestrel Bio Sciences, Thailand). Briefly, 5 µL of dual-labeled PCR product was diluted in 90 µL of assay buffer in a 1.5 mL tube. The diluted PCR product was applied to the loading zone of the PCR-LFA strip and was observed until the strip control line (C line) was visible (approximately 2 min). A positive result for *Aristolochia* occurred when all three T1, T2 and C lines were present, while a negative result was when T1 and C lines were observed. The colored band of each line was evaluated in three stages: pink (++), faint pink (+), and no coloration (−) (Table 2). To confirm a positive result for the presence of *Aristolochia*, the amplicon was purified and subsequently bidirectionally sequenced by gold-standard Sanger sequencing on an ABI 3730XL DNA analyzer.

### Specificity and sensitivity of PCR-LFA

The specificity of PCR-LFA for the detection of *Aristolochia* was evaluated using genomic DNA from nine samples of *Aristolochia* species and four samples of non-*Aristolochia* species that usually substituted for “Krai-Krue”, including *C. pulchellum*, *T. scabra*, *J. sambac*, and *J. adenophyllum* (Table 2). PCR amplification and condition were conducted as mentioned above. PCR amplicons were visualized using PCR*-*LFA (Lot. No. 21F0306, Kestrel Bio Sciences, Thailand) as previously described. The experiment was repeated twice. To confirm the positive result of PCR-LFA from *Aristolochia*, standard PCR amplification with A397F and R502 primers was performed. Then, the PCR amplicon amplified from *A. pothieri* was selected and purified and subsequently bidirectionally sequenced by gold-standard Sanger sequencing on an ABI 3730XL DNA analyzer.

To test the sensitivity of our developed PCR-LFA, genomic DNA from *Aristolochia* at amounts of 2, 1, 0.1 and 0.01 ng was prepared. Each dilution was used as a DNA template for PCR amplification and subjected to LFA. PCR condition was conducted as earlier mentioned. The presence of *Aristolochia* DNA in the LFA system was observed. The remaining PCR products were verified by 1.7% agarose gel electrophoresis for specific and internal control amplicons expected to be 124 bp and 145 bp, respectively. The experiment was repeated twice.

## Conclusions

The nucleotide sequences of the *rbc*L gene from one hundred and seventy-seven accession numbers, including *Aristolochia* and non-*Aristolochia* species, were aligned to seek a nucleotide signature specific to only plants in the genus *Aristolochia*. Surprisingly, the exclusive nucleotide signature in the *rbc*L gene belonging to plants in the genus *Aristolochia* was found. A primer set that targeted the nucleotide signature of *Aristolochia* plants was designed, and duplex PCR coupled with LFA was developed for the rapid and visualized detection of *Aristolochia,* plants responsible for aristolochic acid nephropathy. PCR-LFA was an effective method to detect the existence of *Aristolochia* either in crude drugs or TTM formulae, which could be used in the quality control of herbal medicine and subsequently for consumer safety. From our study, *Aristolochia* crude drugs and *Aristolochia*-containing products were found to be presented in dispensaries. Herbs associated with renal failure and urothelial cancer issues that arise from the use of those plants or plant products should be seriously considered.

## Supplementary Information

Supplementary Information 1.

Supplementary Information 2.

Supplementary Information 3.

Supplementary Information 4.

Supplementary Information 5.

Supplementary Information 6.

Supplementary Information 7.

Supplementary Information 8.

Supplementary Information 9.

## Data Availability

All data generated or analysed during this study are included in this published article [and its supplementary information files].

## References

[1] González F (1999). Inflorescence morphology and the systematics of Aristolochiaceae. Syst. Geogr. Plants.

[2] Do TV, Wanke S, Neinhuis C, Pooma R (2015). *Aristolochia phuphathanaphongiana* sp nov from southwestern Thailand. Nord. J. Bot..

[3] Wagner ST (2014). Major trends in stem anatomy and growth forms in the perianth-bearing Piperales, with special focus on Aristolochia. Ann. Bot..

[4] Heinrich M, Chan J, Wanke S, Neinhuis C, Simmonds MS (2009). Local uses of Aristolochia species and content of nephrotoxic aristolochic acid 1 and 2–a global assessment based on bibliographic sources. J. Ethnopharmacol..

[5] Phumthum M, Balslev H (2020). Anti-Infectious plants of the Thai Karen: a meta-analysis. Antibiotics (Basel).

[6] Tripatara P (2012). The safety of Homnawakod herbal formula containing Aristolochia tagala Cham. in Wistar rats. BMC Complement Altern. Med..

[7] Ministry of Public Health. Edition of Krai-Krue containing registered remedies in *Announcement of the Ministry of Public Health* (ed. Ministry of Public Health Committee) 55 (Royal Thai Government Gazette, 2013).

[8] Dechbumroong P, Aumnouypol S, Denduangboripant J, Sukrong S (2018). DNA barcoding of *Aristolochia* plants and development of species-specific multiplex PCR to aid HPTLC in ascertainment of *Aristolochia* herbal materials. PLoS ONE.

[9] Wu L (2015). An integrated system for identifying the hidden assassins in traditional medicines containing aristolochic acids. Sci. Rep..

[10] Han J, Xian Z, Zhang Y, Liu J, Liang A (2019). Systematic overview of aristolochic acids: nephrotoxicity, carcinogenicity, and underlying mechanisms. Front. Pharmacol..

[11] The U.S. Department of Health and Human Services*. The 15th report on carcinogens* https://ntp.niehs.nih.gov/ntp/roc/content/listed_substances_508.pdf (2021).

[12] Zhang HM (2019). Recognition of the toxicity of aristolochic acid. J. Clin. Pharm. Ther..

[13] Poon SL (2015). Mutation signatures implicate aristolochic acid in bladder cancer development. Genome Med..

[14] Arlt VM, Stiborova M, Schmeiser HH (2002). Aristolochic acid as a probable human cancer hazard in herbal remedies: a review. Mutagenesis.

[15] Jelakovic B (2015). Renal cell carcinomas of chronic kidney disease patients harbor the mutational signature of carcinogenic aristolochic acid. Int. J. Cancer.

[16] Ekar T, Kreft S (2019). Common risks of adulterated and mislabeled herbal preparations. Food Chem. Toxicol..

[17] Vuthithammavech, V. *Encyclopedia of Herbs*. 618 (O.S. Printing House, 1997).

[18] Bureau of Drug and Narcotic. *Thai herbal pharmacopoeia 2021.* (Department of Medical Sciences, 2021).

[19] Draghia, L.P. et al. Aristolochic acid I: an investigation into the role of food crops contamination, as a potential natural exposure pathway. *Environ Geochem Health* (2021). 10.1007/s10653-021-00903-4 33796971

[20] Agrawal P, Laddha K (2017). Development of validated high-performance thin layer chromatography for quantification of aristolochic acid in different species of the Aristolochiaceae family. J. Food Drug. Anal..

[21] Michl J (2016). LC-MS- and (1)H NMR-based metabolomic analysis and *in vitro* toxicological assessment of 43 *Aristolochia* species. J. Nat. Prod..

[22] Graff, A. & Upton R. Characterization of selected plants that may contain or be adulteranted with aristolochic acid (American Herbal Pharmacopoeia Scotts Valley, CA; 2006).

[23] Raeisossadati MJ (2016). Lateral flow based immunobiosensors for detection of food contaminants. Biosens. Bioelectron..

[24] Wong YL, Wong KL, Shaw PC (2015). Rapid authentication of Cordyceps by lateral flow dipstick. J. Pharm. Biomed. Anal..

[25] Thongkhao K, Tungphatthong C, Phadungcharoen T, Sukrong S (2020). The use of plant DNA barcoding coupled with HRM analysis to differentiate edible vegetables from poisonous plants for food safety. Food Control.

[26] Guo M, Jiang W, Yu J, Pang X (2020). Investigating the authenticity of Ophiopogonis Radix and its Chinese patent medicines by using a nucleotide signature. J. Ethnopharmacol..

[27] Sgamma T (2017). DNA barcoding for industrial quality assurance. Planta Med..

[28] Sgamma T, Masiero E, Mali P, Mahat M, Slater A (2018). Sequence-specific detection of *Aristolochia* DNA – a simple test for contamination of herbal products. Front Plant. Sci..

[29] Pecchia S, Da Lio D (2018). Development of a rapid PCR-Nucleic Acid Lateral Flow Immunoassay (PCR-NALFIA) based on rDNA IGS sequence analysis for the detection of Macrophomina phaseolina in soil. J. Microbiol. Methods.

[30] Yamamuro T (2018). Development of simple and accurate detection systems for *Cannabis sativa* using DNA chromatography. Forensic Sci Int.

[31] Amalina ZN (2021). Nucleic acid-based lateral flow biosensor for *Salmonella typhi* and *Salmonella paratyphi*: a detection in stool samples of suspected carriers. Diagnostics (Basel).

[32] Brown AC (2017). Kidney toxicity related to herbs and dietary supplements: online table of case reports. Part 3 of 5 series. Food Chem. Toxicol..

[33] Bureau of Drug and Narcotic. *Thai herbal pharmacopoeia 2020.* (Department of Medical Sciences, 2020).

[34] Chen X (2013). A fast SNP identification and analysis of intraspecific variation in the medicinal *Panax* species based on DNA barcoding. Gene.

[35] Han J (2016). An authenticity survey of herbal medicines from markets in China using DNA barcoding. Sci. Rep..

[36] Anerao J, Jha V, Desai N (2017). Optimization of DNA extraction methods from *Garcinia* species for ISSR-PCR, RAPD-PCR and DNA barcoding. Asian J. Biotechnol..

[37] Thongkhao K (2020). Integrative approaches for unmasking hidden species in herbal dietary supplement products: What is in the capsule?. J. Food Compos. Anal..

[38] Jiang N (2019). Lateral and vertical flow assays for point-of-care diagnostics. Adv Healthc. Mater..

[39] Dong SWWX, Shang MY, Ma CM, Zhang SX, Li XW, Xu MJ, Cai SQ, Namba T (2010). Sesquiterpene and aristolochic acid derivatives from *Thottea hainanensis*. Helv. Chim. Acta.

[40] Ankola, K., Mahadevegowda, L., Melichar, T., & Boregowda, M. H. DNA barcoding: nucleotide signature for identification and authentication of livestock in Advances in Animal Genomics. (ed. Mondal, S. & Singh, R.L) 299–308 (Academic Press, 2021).

[41] Dong W (2014). Discriminating plants using the DNA barcode *rbc*Lb: an appraisal based on a large data set. Mol. Ecol. Resour..

[42] Devey DS, Chase MW, Clarkson JJ (2009). A stuttering start to plant DNA barcoding: microsatellites present a previously overlooked problem in non-coding plastid regions. Taxon.

[43] Ebihara A, Nitta JH, Ito M (2010). Molecular species identification with rich floristic sampling: DNA barcoding the pteridophyte flora of Japan. PLoS ONE.

[44] Safenkova IV (2020). Key significance of DNA-target size in lateral flow assay coupled with recombinase polymerase amplification. Anal. Chim. Acta.

[45] Xin T (2018). Biomonitoring for traditional herbal medicinal products using DNA metabarcoding and single molecule, real-time sequencing. Acta Pharm. Sin. B.

