# Supplementary material for: A PCR-lateral flow immunochromatographic assay (PCR-LFA) for detecting Aristolochia species, the plants responsible for aristolochic acid nephropathy
Source: Sci Rep. 2022 Jul 16;12:12188. doi: 10.1038/s41598-022-16528-1 (PMC9288547; doi:10.1038/s41598-022-16528-1)

**Supplementary Fig. S1** Blast results of our designed primers (A397F and R502) and PCR amplicon against NCBI database.

**A397F primer**
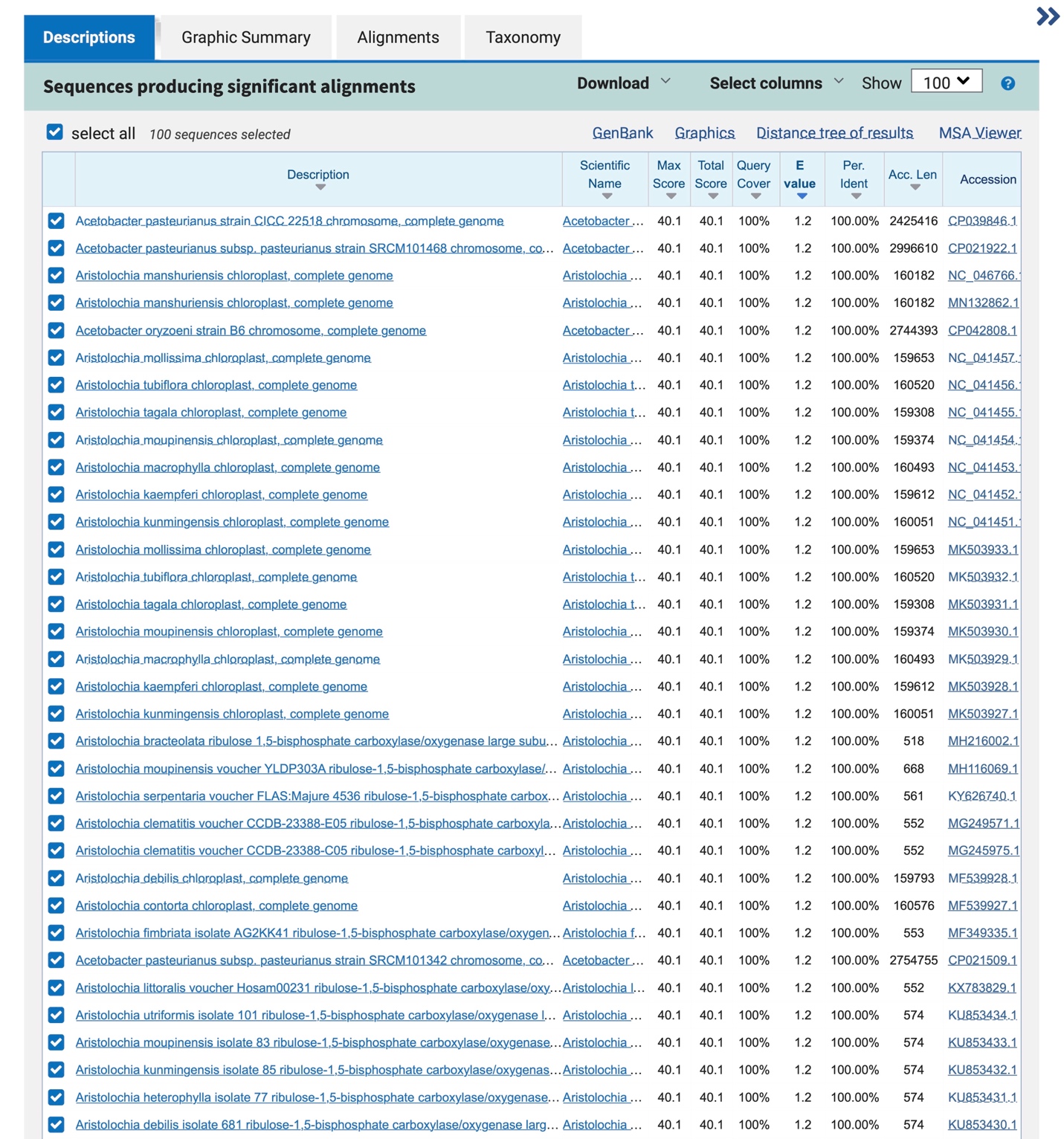


**R502 primer**


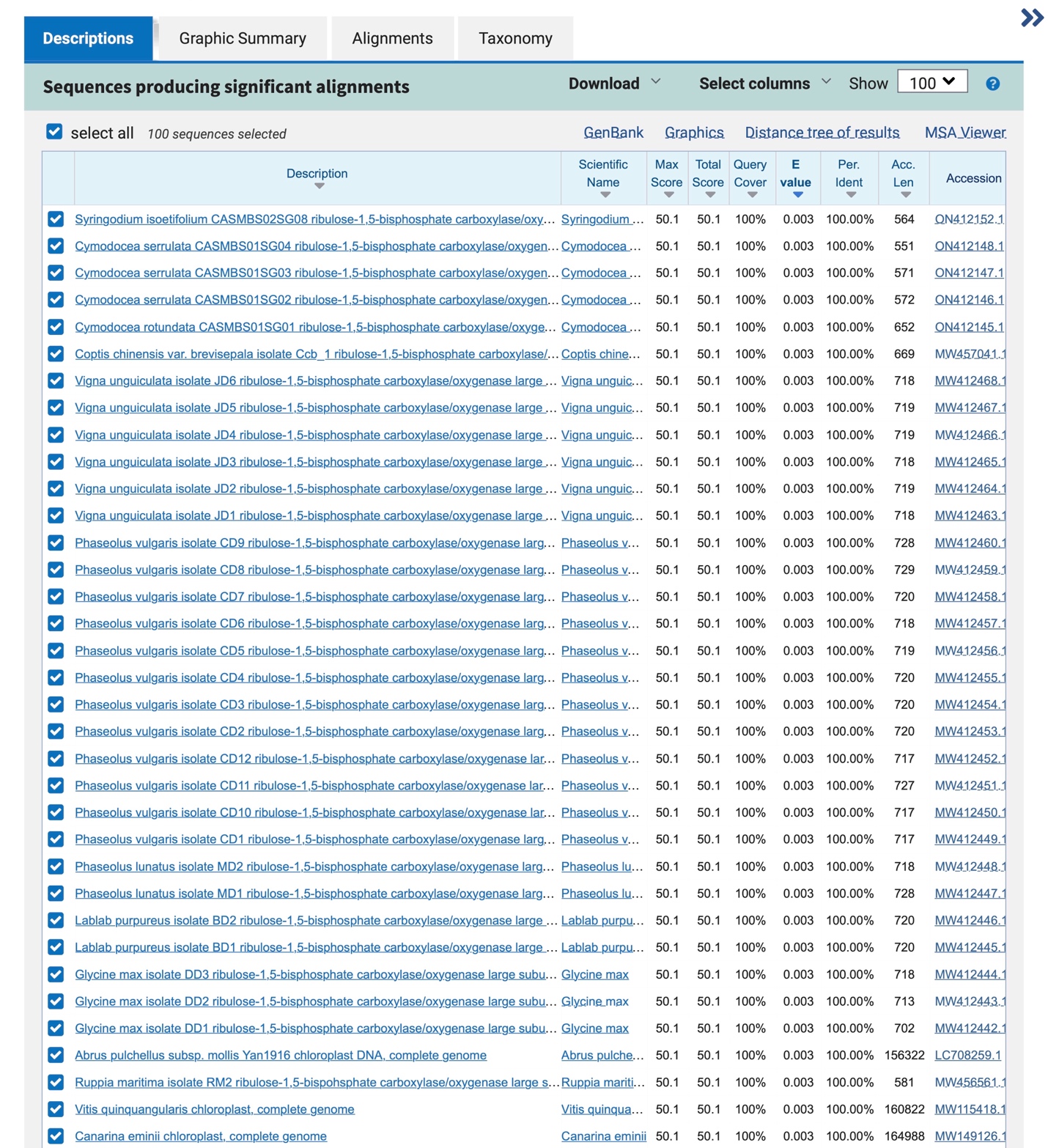


**PCR amplicon amplified from A397F and R502 primers.**


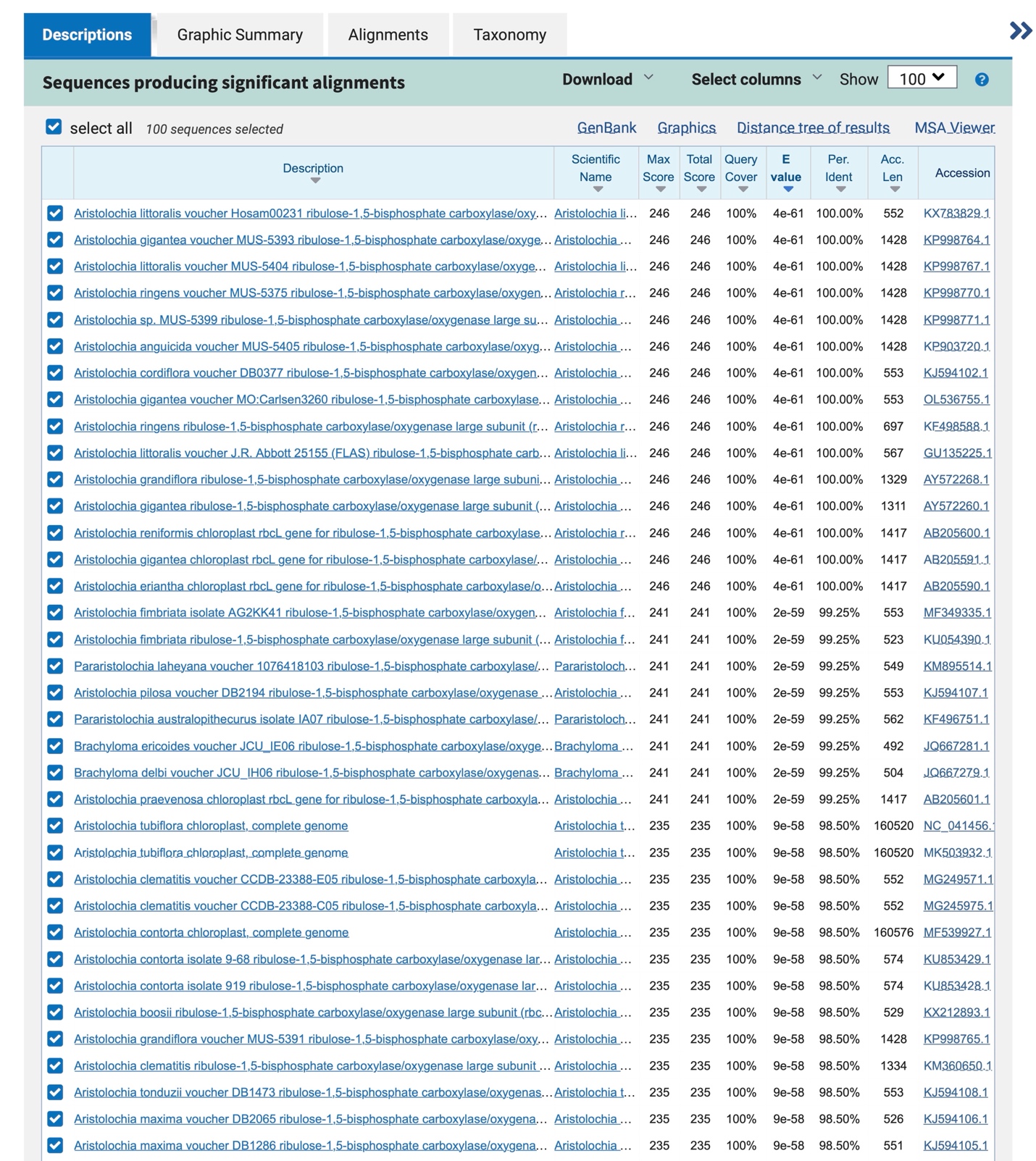

Supplement: Supplementary file 2 — Supplementary Information 2. [file 41598_2022_16528_MOESM2_ESM.docx]
