# Supplementary material for: A PCR-lateral flow immunochromatographic assay (PCR-LFA) for detecting Aristolochia species, the plants responsible for aristolochic acid nephropathy
Source: Sci Rep. 2022 Jul 16;12:12188. doi: 10.1038/s41598-022-16528-1 (PMC9288547; doi:10.1038/s41598-022-16528-1)
Supplement: Supplementary file 3 — Supplementary Information 3. [file 41598_2022_16528_MOESM3_ESM.docx]

**Supplementary Fig. S2**


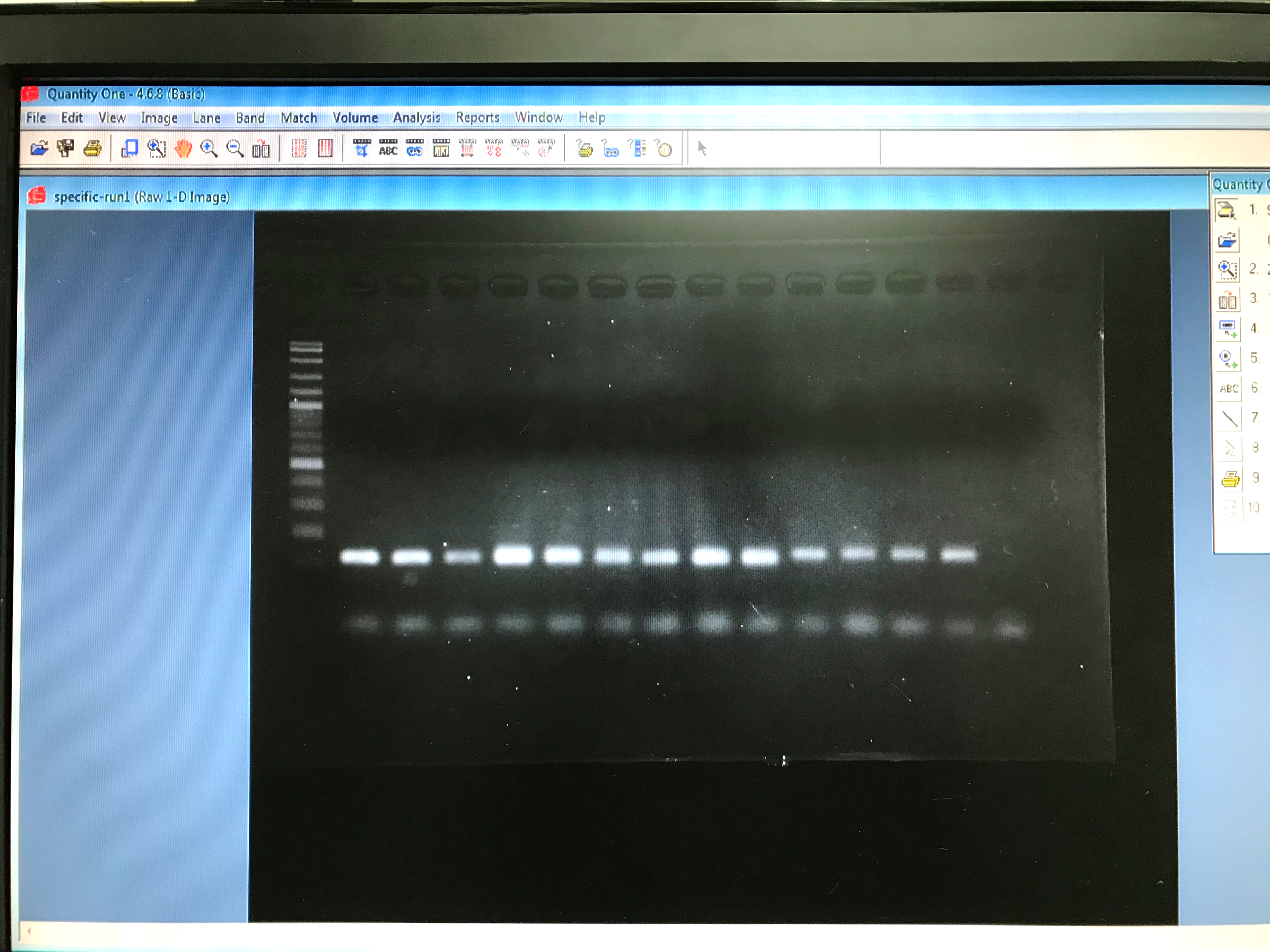


**M 1 2 3 4 5 6 7 8 9 10 11 12 13 14**

1 kb

500 bp

Primer dimer

PCR amplicons

100 bp

**Raw image of Fig. 5A**

Image of PCR amplicons as detected by 1.7% agarose gel electrophoresis. **1**: *A. pierrei*, **2**: *A. pothieri*, **3**: *A. tagala*, **4**: *A. gigantea.* **5**: *A. grandiflora*, **6**: *A. cambodiana*, **7**: *A. littoralis,* **8**: *A. ringens*, **9**: *A. tentaculata*, **10**: *C.* *pulchellum*, **11**: *T.* *scabra*, **12**: *J. sambac*, **13**: *J.* *adenophyllum*, **14**: no-DNA template as a control reaction.


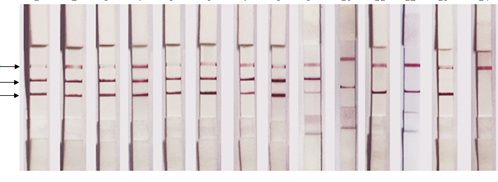


**1 2 3 4 5 6 7 8 9 10 11 12 13 14**

T1 line

T2 line

C line

**Raw image of Fig. 5B**

Specificity of the PCR*-*LFA for the detection of PCR amplicons generated with a set of primers, A397F, C357F and R502.

(B) Specificity of the PCR*-*LFA on samples; **1**: *A. pierrei*, **2**: *A. pothieri*, **3**: *A.acuminata*, **4**: *A. gigantea.* **5**: *A. grandiflora*, **6**: *A. cambodiana*, **7**: *A. littoralis,* **8**: *A. ringens*, **9**: *A. tentaculata*, **10**: *C.* *pulchellum*, **11**: *T.* *scabra*, **12**: *J. sambac*, **13**: *J.* *adenophyllum*, **14**: no-DNA template as a control reaction.

C line: strip control line, T1 line: an internal control line, T2 line: *Aristolochia* line

**1 2 3 4 5**


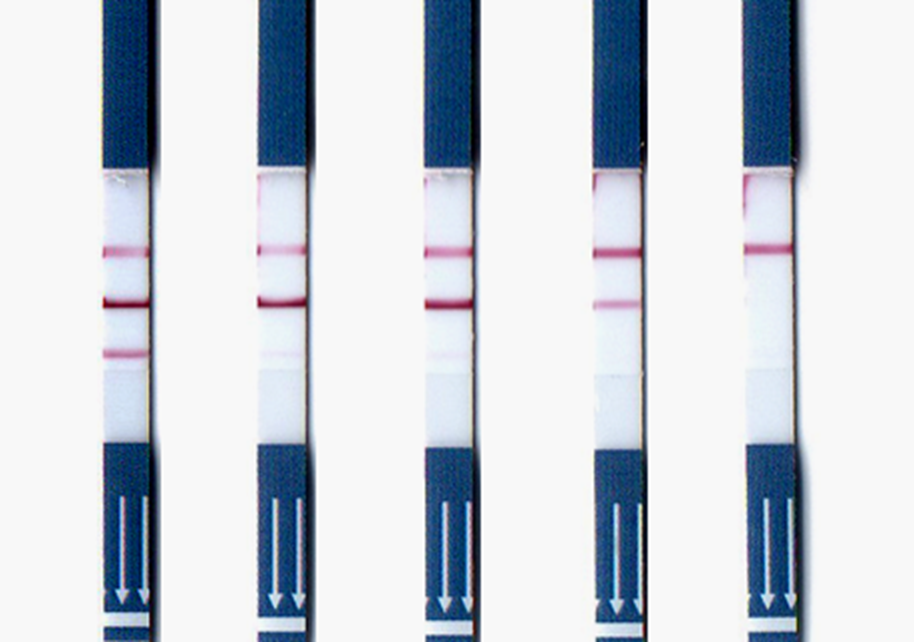


T2 line

C line

T1 line

**Raw image of Fig. 5C**

Sensitivity of the *Aristolochia* PCR*-*LFA. Template DNA: 2 ng (lane 1), 1 ng (lane 2), 0.1 ng (lane 3) and 0.01 ng (lane 4). NTC: no-DNA template (lane 5).

C line: strip control line, T1 line: an internal control line, T2 line: *Aristolochia* line
