# Supplementary material for: A PCR-lateral flow immunochromatographic assay (PCR-LFA) for detecting Aristolochia species, the plants responsible for aristolochic acid nephropathy
Source: Sci Rep. 2022 Jul 16;12:12188. doi: 10.1038/s41598-022-16528-1 (PMC9288547; doi:10.1038/s41598-022-16528-1)

**Supplementary Fig. S3** Confirmation of nucleotide sequences from PCR amplicons amplified from an *Aristolochia* plant sample, *A. pothieri.*

A) Electropherogram of *Aristolochia* PCR amplicon. The A397F primer was used as the sequencing primer.

B) Nucleotide sequence read from electropherogram

TTCCTACCGCTTATATCAAAACTTTCCAAGGCCCTCCCCACGGTATCCAAGTTGAGAGAGATAAATTGAACAAGTATGGTCGTCCAA

C) Nucleotide Blast result from read sequences indicated *Aristolochia* species


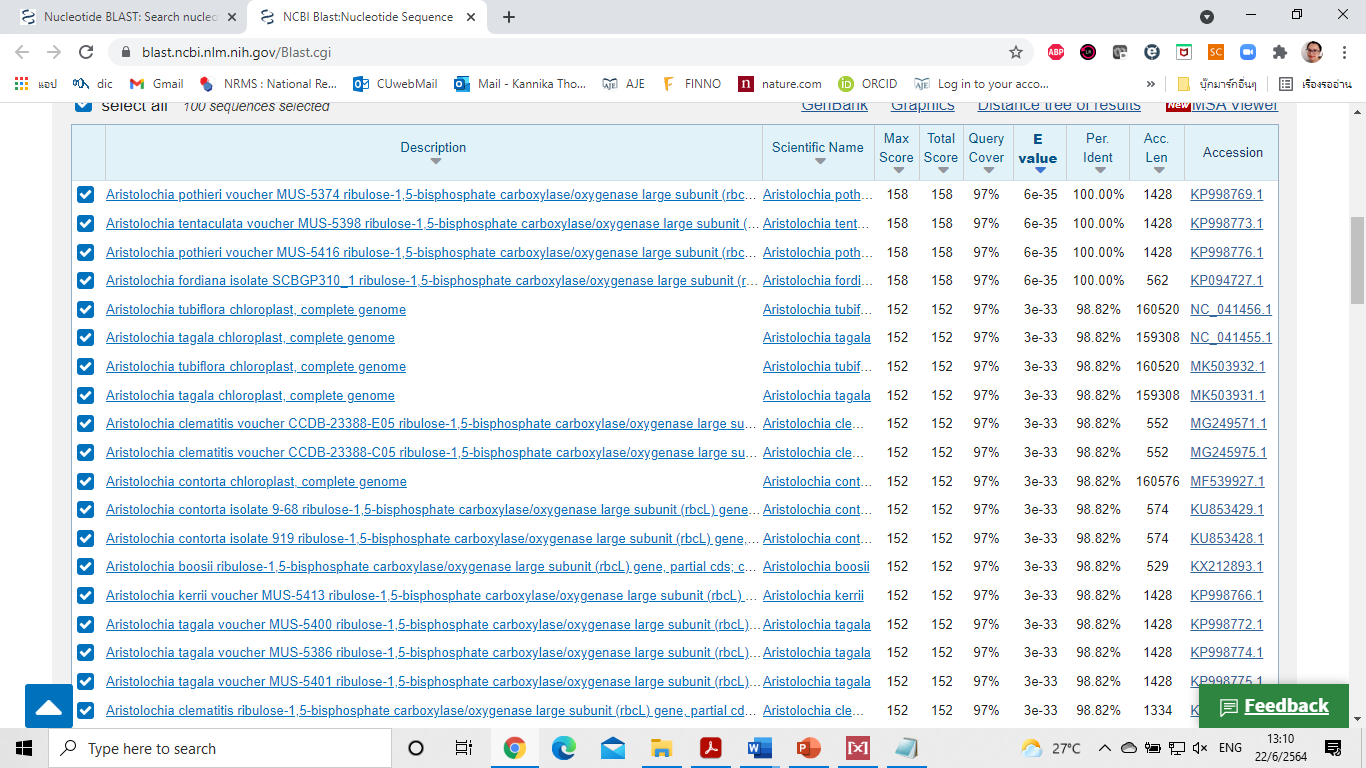

Supplement: Supplementary file 4 — Supplementary Information 4. [file 41598_2022_16528_MOESM4_ESM.docx]
