# Supplementary material for: A PCR-lateral flow immunochromatographic assay (PCR-LFA) for detecting Aristolochia species, the plants responsible for aristolochic acid nephropathy
Source: Sci Rep. 2022 Jul 16;12:12188. doi: 10.1038/s41598-022-16528-1 (PMC9288547; doi:10.1038/s41598-022-16528-1)
Supplement: Supplementary file 5 — Supplementary Information 5. [file 41598_2022_16528_MOESM5_ESM.docx]

**Supplementary Fig. S4**

**+ - C1 C2 C3 C4 C5 C6 C7 C8 C5R NTC**


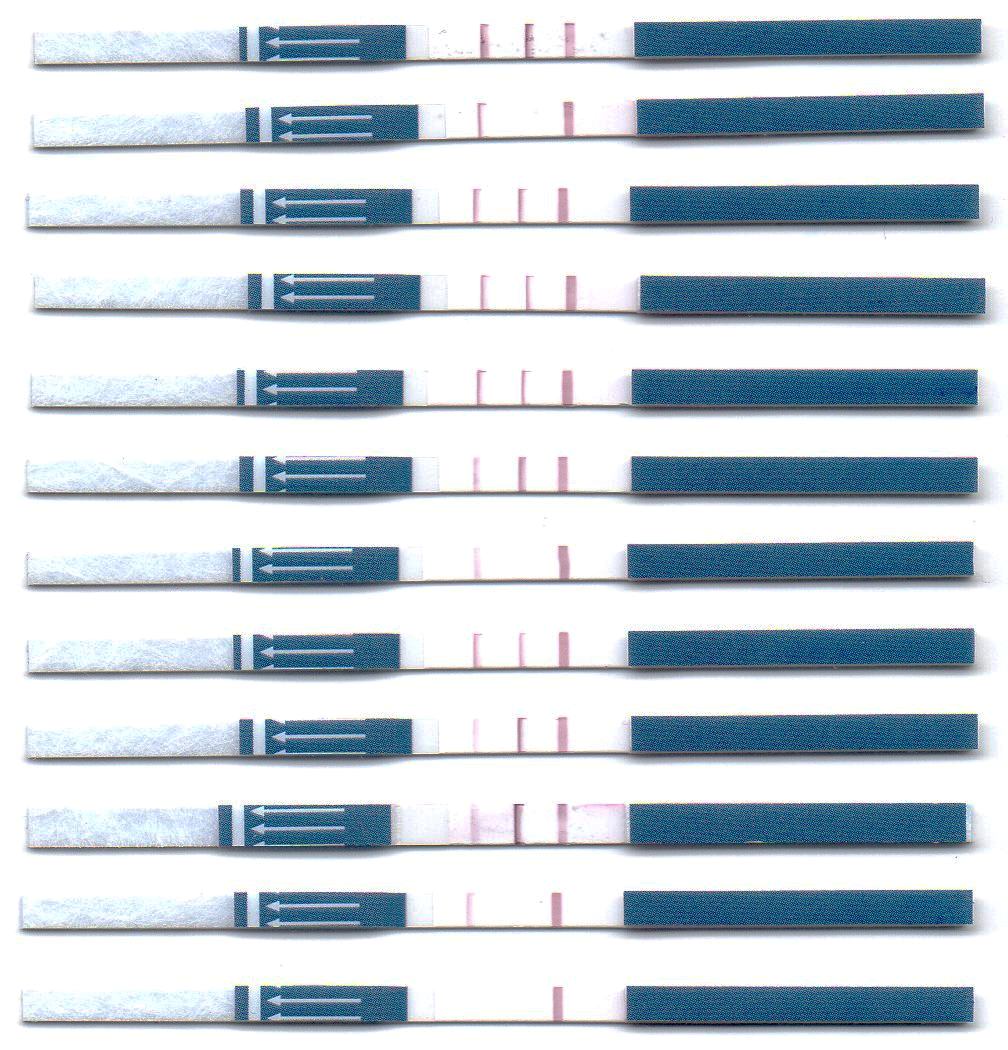


T2 line

C line

T1 line

**Raw image of Fig. 6A**

Testing the existence of *Aristolochia* spp*.* in herbal products with the PCR- LFA method. (A) crude drugs. +: positive control (SS-A0716 *Aristolochia* *pierrei*), -: negative control (SS-A0726 *Jasminum sambac*), C1-C8: crude drug no. 1-8, C5R: crude drug no. 5 (repeat). NTC: no-DNA template as a control reaction.

**+ - F0 F1 F2 F3 F4 NTC**


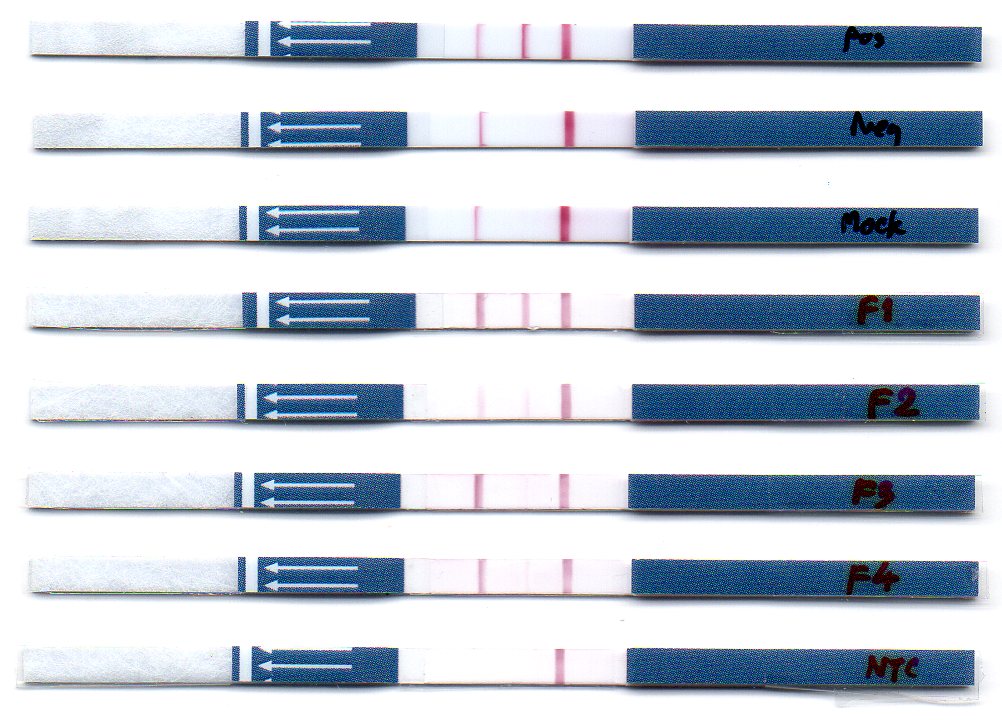


C line

T2 line

T1 line

**Raw image of Fig. 6B**

Testing the existence of *Aristolochia* spp*.* in herbal products with the PCR- LFA method. (B) herbal formulae. +: positive control, -: negative control, F0: laboratory-made formulae (YMVT), F1-4: herbal formula no. 1-4, NTC: no-DNA template as a control reaction.
