# Supplementary material for: A PCR-lateral flow immunochromatographic assay (PCR-LFA) for detecting Aristolochia species, the plants responsible for aristolochic acid nephropathy
Source: Sci Rep. 2022 Jul 16;12:12188. doi: 10.1038/s41598-022-16528-1 (PMC9288547; doi:10.1038/s41598-022-16528-1)

**Supplementary Fig. S5** Electropherograms of PCR amplicons amplified from selected samples (F1, C7 and C8) after samples obtained positive results from the PCR-LFA method. Sequencing for confirmation of *Aristolochia* plants was conducted by using reverse primers. Arrows indicate nucleotides (position 197 of the *rbc*L gene) specific to *Aristolochia* spp.

1. Electropherogram of an *Aristolochia*-specific amplicon from the F1 product.


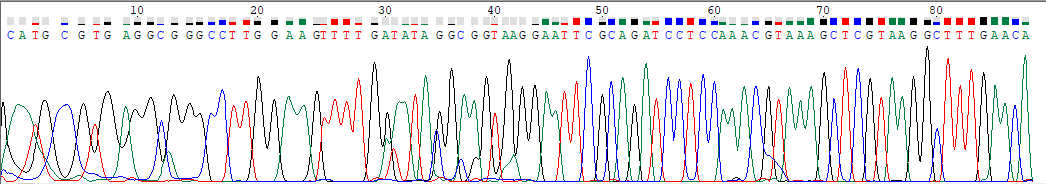


1. Electropherogram of an *Aristolochia*-specific amplicon from C7.

1. Electropherogram of *Aristolochia*-specific amplicon from C8.
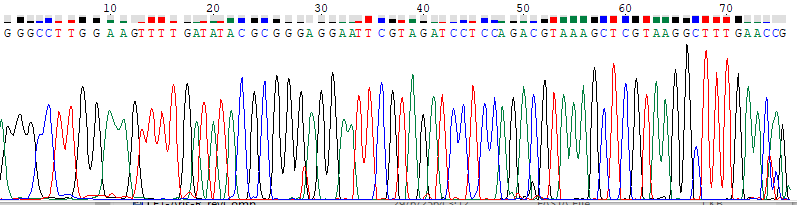

Supplement: Supplementary file 6 — Supplementary Information 6. [file 41598_2022_16528_MOESM6_ESM.docx]
